# Preparation of boron nitride nanosheets *via* polyethyleneimine assisted sand milling: towards thermal conductivity and insulation applications†

**DOI:** 10.1039/d1ra05878a

**Published:** 2021-11-29

**Authors:** Bing Wang, Haifeng Ji, Xiaojie Zhang, Xiongwei Qu

**Affiliations:** Hebei Key Laboratory of Functional Polymers, Department of Polymer Materials and Engineering, Hebei University of Technology 8 Guangrong Street Tianjin 300130 P. R. China xwqu@hebut.edu.cn zhangxj@hebut.edu.cn

## Abstract

Hexagonal boron nitride (h-BN) is often used as a filler in polymer composites due to its good thermal conductivity and insulation properties. However, the compatibility between h-BN and the matrix limits its application areas. To overcome this issue, a combination of mechanical liquid phase exfoliation and chemical interfacial modification was adopted in this work. Polyethyleneimine (PEI) was used as the exfoliation reagent to prepare PEI-functionalized h-BN nanosheets, denoted as PEI@BNNS. Thermoplastic polyurethane (TPU) composites with different contents of h-BN and PEI@BNNS which were recorded as h-BN/TPU and PEI@BNNS/TPU were successfully prepared through a hot-pressing process, respectively. The results show that PEI@BNNS/TPU composites have better in-plane thermal conductivity while maintaining insulation, and with the content of 5 wt% PEI@BNNS, the in-plane thermal conductivity of the PEI@BNNS/TPU composite is up to 0.61 W m^−1^ K^−1^, which is three times that of pure TPU (0.22 W m^−1^ K^−1^).

## Abstract 2

The PEI-grafted boron nitride nanosheets were successfully prepared *via* sand-milling process, which were doped into thermoplastic polyurethane matrix for better in-plane thermal conductivity while maintaining insulation properties.

## Graphical abstract

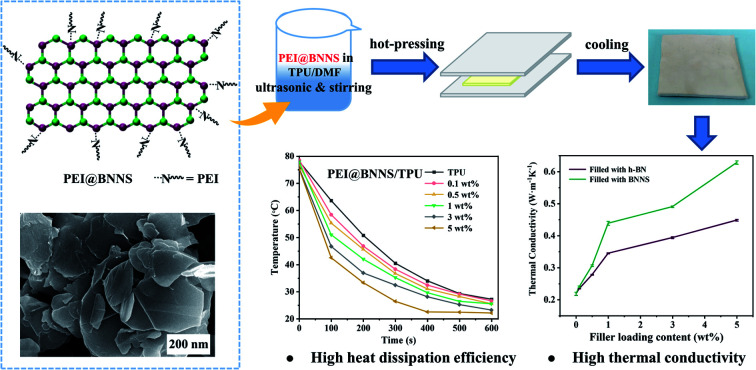

## 1 Introduction

Electronic components are developing in the direction of high frequency, high speed, high power and high integration. Even so, these will cause the internal heat of the device to accumulate rapidly, which greatly affects the stability and service life of electronic products. Therefore, it is urgent to use high thermal conductivity materials for thermal management to remove excess heat from the electronic equipment to the surrounding environment.^1,2^ Generally speaking, the heat dissipation process mainly includes four stages: (a) heat transfer within the equipment itself; (b) heat transfer from equipment to radiators; (c) heat transfer of the radiator itself; (d) heat transfer from the radiator to the surrounding environment, which the heat transfer between the equipment and the radiator is generally considered to be the limiting heat transfer stage.^3^ Traditional thermal conductive materials are mainly including metal materials, metal oxides, metal nitrides and some inorganic non-metallic materials. Still and all, these materials have some poor properties such as easy corrosion, easy conductivity and high cost, which limit their development in the fields of electronics.^4,5^

Polymer materials are light, easy to process, and have good mechanical properties, electrical insulation, chemical stability and fatigue resistance, which have broad development prospects and play an important role in the preparation of electronic packaging materials with thermal conductivity, where the unique micro phase separation structure of thermoplastic polyurethane (TPU) makes it have good wear resistance, high process ability, excellent chemical stability and good mechanical properties. In the process of synthesis, different properties can be obtained by controlling the structure and proportion of soft and hard segment materials, which are kind of high plasticity polymer materials with wide application prospects.^6–10^ However, the thermal conductivity of polymer material is relatively low (about 0.2 W m^−1^ K^−1^), where the addition of the thermal conductive fillers is an effective solution to improve thermal conductivity. The common thermal conductive fillers are mainly divided into metal materials (such as Al, Ag, Cu, *etc.*),^11–13^ inorganic non-metal materials (such as Al_2_O_3_, AlN, BN, *etc.*),^14–18^ carbon-based materials (such as graphite, carbon nanotubes, carbon fiber, *etc.*).^19–21^

Hexagonal boron nitride (h-BN) is a graphene-like layered material, commonly known as “white graphite”,^22^ which still has good lubrication performance at high temperatures, and the breakdown strength can reach 35 V·μm^−1^.^23^ At the same time, h-BN is chemically stable and chemically inert to acids and all molten metals.^24^ In addition, its wide band gap of 5.9 eV gives it excellent electrical insulation properties such as high resistivity, low dielectric constant and low dielectric loss.^25^ These excellent properties make h-BN often to be used as filler to improve the thermal conductivity of the composites. Even so, unless the filler content is very high, the enhancement efficiency is usually unsatisfactory. One of the most important reasons is the high interfacial thermal resistance (ITR) produced by the inherent interface between the phases in the materials,^2–4,26^ which the heat consumption rate per unit mass of filler should follow the trend of 2D > 1D > 0D.^27–29^ Thus, there are numerous preparation methods of 2D layered BNNS (boron nitride nanosheets) have been reported,^30–40^ which have higher chemical and thermal stability as well as unique insulation properties compared with few layers of graphite.^41^ However, owing to there are almost no active groups on BNNS so that the structure of BNNS is stable, and the adhesion between BNNS and polymer matrix is poor, so it is necessary to carry out a surface modification to improve the compatibility between BNNS and polymer matrix.^42^

In this paper, the combination of mechanical liquid phase exfoliation and chemical modification was employed to peel h-BN into BNNS by using PEI as sand grinding auxiliary. h-BN was successful exfoliated, and relatively thin BNNS with PEI grafted-on were obtained due to the intermolecular interaction between B atom in h-BN and amino groups in PEI. Subsequently, TPU based composites with PEI@BNNS as filler were prepared by means of hot-pressing, which exhibits good insulation and higher in-plane thermal conductivity than that of h-BN/TPU composites. This approach may provide more information for the construction of heat conduction network and preparation of insulating thermally conductive composites materials.

## 2 Experimental methods

### 2.1 Materials

Polyethyleneimine (50% aqueous solution) with a molecular weight of 70 000 was purchased from Shanghai Aladdin Biochemical Technology Co., Ltd. Hexagonal boron nitride (h-BN) was purchased from Shandong Qingzhou Materials Technology Co., Ltd. with an average particle size of 5 μm (purity > 99%). Thermoplastic polyurethane elastomer (TPU), made from BASF in Germany, the brand is 1180A10, in order to avoid the influence of water on the system, it is dried in the oven at 80 °C for 8 h before using. *N*,*N*-Dimethyl formamide (analytically pure), purchased from Tianjin Concord Technology Co., Ltd. Anhydrous ethanol (analytically pure), purchased from Tianjin Fuchen Chemical Reagent Factory.

### 2.2 Preparation of PEI@BNNS

Firstly, 200 mL PEI aqueous solution with a mass fraction of 2 wt% was prepared, and stirred it on a magnetic stirrer for 30 min until the viscous PEI was completely dissolved in deionized water to form a colorless and transparent aqueous solution. Then 2 g h-BN powder and 200 g zirconia sand grinding ball with a diameter of 2 mm were weighed. The above three kinds of materials were added into the stainless steel sanding tank in turn for sanding, which the rotational speed and the sanding time are 2000 rpm and 8 h, respectively. After sanding, sanding beads were removed with a 20-mesh sieve and the suspension was collected into a beaker to conduct ultrasonic (80 W) for 2 h by using an ultrasonic cleaner. After ultrasonic, the suspension was centrifuged with the rotation speed of 3000 rpm for 15 min, and collect the supernatant. Finally, the collected supernatant was centrifuged at 10 000 rpm for 25 min and freeze-dried for 24 h to obtain PEI@BNNS.

### 2.3 Preparation of TPU composites

PEI@BNNS/TPU composites were prepared by solution blending and hot-pressing, where the mass fractions of PEI@BNNS are 0.1 wt%, 0.5 wt%, 1 wt%, 3 wt%, and 5 wt%, respectively. The specific preparation process is as follows: first, the TPU was dried in an oven at 80 °C for 6 hours to remove moisture. Take 100 mL of DMF and add PEI@BNNS with different contents with 1 h of ultrasound and pour into a four-necked flask, then 10 g TPU particles was taken into the flask with mechanically stir for 8 h in a water bath at 80 °C to obtain a viscous liquid, namely PEI@BNNS/TPU composites. Next, pour it into 800 mL of ethanol to co-precipitate to remove DMF, and the flocculent mixture was obtained by standing. After vacuum filtration, the flocculent mixture was dried in a vacuum chamber at 50 °C for 24 h until its weight did not change. PEI@BNNS/TPU composites after drying was put into the mold at 180 °C for 8 min under normal pressure, then hot press at 10 MPa pressure for 10 min, finally cooled down for 10 min to obtain the PEI@BNNS/TPU composites. The h-BN/TPU composites and pure TPU were prepared as the control group according to the above preparation methods.

### 2.4 Structural characterizations

Fourier transform infrared spectroscopy (FTIR, Vector-22, Bruker, Germany) of h-BN, PEI@BNNS and PEI were recorded. The spectral range was 400–4000 cm^−1^ with a resolution of 4 cm^−1^. Thermal gravimetric analysis (TGA, Q600, TA Instrument, USA) was used to analyze the thermal weight loss of h-BN and PEI@BNNS. The test temperature is 30–800 °C, the heating rate is 10 °C min^−1^, and the nitrogen flow rate is 100 mL min^−1^ to ensure that the volatiles can diffuse in time. XRD patterns of h-BN and TPU composites were collected using X-ray diffraction (XRD, D8 Advance, Bruker AXS, Germany) at scan steps of 6° min^−1^ from 10° to 80°. The elemental composition of PEI@BNNS was analyzed by X-ray photoelectron spectroscopy (XPS, ESCALAB 250Xi, Thermo Fisher Scientific, USA). Zeta potentials of h-BN and PEI@BNNS were measured with potential analyzer (Nano-ZS90, Malvern, UK). Scanning electron microscope (SEM, Nova Nano SEM 450, FEI, USA) was used to observe the morphology of h-BN, PEI@BNNS and TPU composites. Before the morphology observation, all the specimens were sprayed with gold. Atomic force microscope (AFM, MultiMode8, Bruker, USA) was used to test the diameter and thickness of PEI@BNNS with ScanAsyst Mode. The morphology of PEI@BNNS was observed with the transmission electron microscope (TEM, JEM 2100F, Japan Electronics Corporation).

### 2.5 Performance

Dynamic mechanical properties were determined by a dynamic mechanical analyzer (DMA, Tritec 2000, Triton Technology, UK), which the test temperature range is from −80 °C to 40 °C at the rate of 3 °C min^−1^. The transient hot wire technology was used to measure the in-plane thermal conductivity of TPU composites samples by the thermal conductivity measuring instrument (TC 3000, Xi'an Xiaxi Electronic Technology Co., Ltd., China). In this method, the linear heat source is placed between two uniform and smooth tested samples, and it is heated with a constant voltage current when the two samples are in thermal equilibrium, then it will transfer the heat to the surrounding medium (the tested sample). The in-plane thermal conductivity of the tested sample determines the rate of heat transfer, which in turn reflects the rate of temperature rise to the linear heat source. Therefore, the in-plane thermal conductivity *k* of the tested TPU composites can be calculated by the following formula:
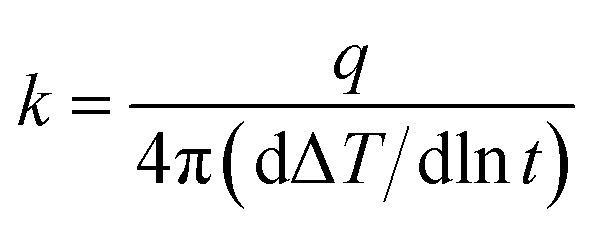
where *q* is the heat generated by the hot wire per unit time and unit length, Δ*T* is the temperature change of the hot wire, and *t* is the time of measurement.

The microcomputer-controlled electronic universal testing machine (CMT6104, Shenzhen Sansixin Co., Ltd., China) was used to test the mechanical properties of TPU composites with the tensile rate is 50 mm min^−1^. The surface resistivity and volume resistivity of composites were tested by using the high resistance meter (ZC36, Shanghai No. 6 Electric Meter Factory, China). The infrared thermal imager (DS-2TPH10-3AUF, Hangzhou Hikvision Digital Technology Co., Ltd., China) was used to record the surface temperature changes of the composites during the cooling process.

## 3 Results and discussion

### 3.1 Characterizations of PEI@BNNS

**Fig. 1:**
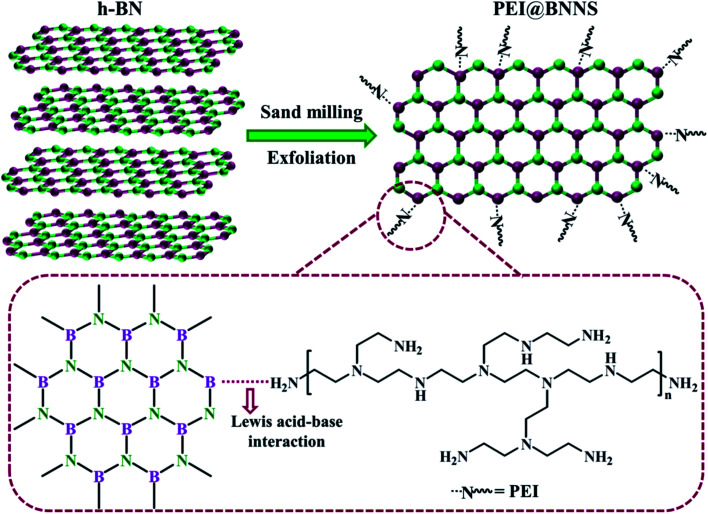
The exfoliation process of h-BN.

**Fig. 2:**
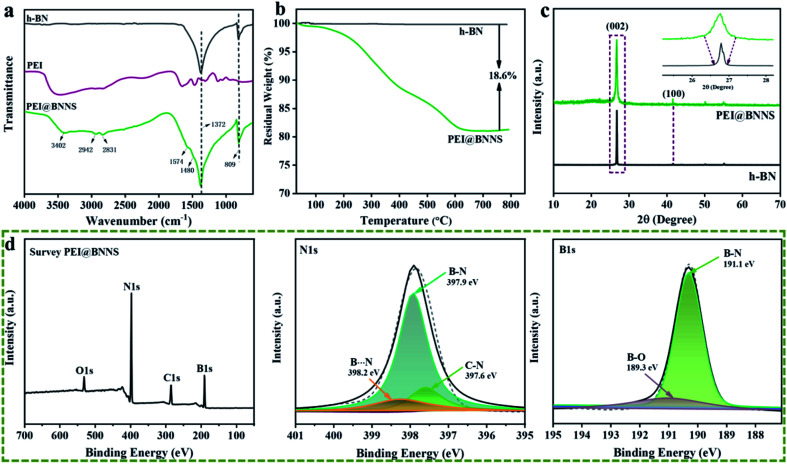
(a) FT-IR spectra of h-BN, PEI@BNNS and PEI. (b) TGA curves of h-BN and PEI@BNNS. (c) XRD patterns of h-BN and PEI@BNNS. (d) XPS survey spectra of PEI@BNNS with the high resolution N 1s and B 1s peak-fitting curves.

The PEI@BNNS was prepared by sanding milling of h-BN with PEI as sanding additives, which was illustrated in the Fig. 1a, and the structural changes of h-BN and PEI@BNNS were characterized by FTIR, TG, XRD and XPS. As shown in Fig. 2a, the FTIR spectra of h-BN and PEI@BNNS both have absorption peaks near 1372 cm^−1^ and 809 cm^−1^, which corresponding to the in-plane stretching vibration peak and the out-of-plane bending vibration peak of B–N,^43^ respectively. In addition to the characteristic absorption peaks of h-BN, some new absorption peaks appeared in curve of PEI@BNNS. The broad peak near 3402 cm^−1^ was derived from –NH_2_, and the peaks around 2942 cm^−1^ and 2831 cm^−1^ were assigned to the stretching vibration of –CH_2_ as well as the peak near 1480 cm^−1^ due to the in-plane bending vibration of –CH_2_. Besides, the N–H bending vibration peaks of primary and secondary amine was at 1574 cm^−1^. These new absorption peaks preliminarily show that PEI moieties are successfully grafted or coated on h-BN. The thermal stability of h-BN and PEI@BNNS were characterized by thermal gravimetric analysis, respectively, as shown in Fig. 2b. PEI@BNNS starts to degrade at about 300 °C and then experience major weight loss between 300 °C with 600 °C while h-BN still has no decomposition at up to 800 °C, which was due to the removal of PEI with a grafting rate of 18.6% on h-BN.^28^

To further prove the effect of sanding on the microstructure of PEI@BNNS, the XRD patterns of h-BN and PEI@BNNS were obtained, respectively. As shown in Fig. 2c, the characteristic peaks of h-BN can still be seen in the PEI@BNNS, which shows that the strong mechanical sanding force does not destroy the crystal structure of h-BN. The characteristic diffraction peak of h-BN at 2*θ* = 26.5° corresponds to the (002) lattice plane which has high peak intensity and sharp peak shape. After sanding, the peak intensity of PEI@BNNS on the (002) crystal plane has been reduced to a certain degree. This is because the normal impact force and tangential shear force^29^ generated when the sand grinding ball collides with layered crystal h-BN overcome the interlayer force of h-BN, thus reducing the thickness and crystallinity of h-BN. Crystal grain size can be characterized by the full width half maximum (FWHM)^19,44^ which gradually widens with the decrease of grain size. Compared with h-BN, the FWHM of PEI@BNNS was broadened when the sanding time is 8 h, which indicates that the number of BN layers decreases.

XPS test was performed to further understand the surface chemical compositions of PEI@BNNS as shown in Fig. 2d. The C 1s, O 1s, B 1s and N 1s elements are seen both in the survey spectra of PEI@BNNS.^45^ Notably, excepting for the peaks at 397.6 eV (C–N) and 397.9 eV (B–N), a new peak was observed at 398.2 eV (B⋯N) from the N 1s spectra of PEI–BNNS, which corresponds to the intermolecular interaction between B atom in BN and the amino groups in PEI.^46^ The high resolution B 1s spectra of PEI@BNNS can be divided into two major peaks located at 191.1 eV and 189.3 eV, which are correspond to the B–N and the, while the B–O bond maybe caused by the ultrasonic process.^47^ Besides, three peaks from PEI in the N 1s spectra of PEI@BNNS can be seen in Fig. S1.†^44^ Zeta potential is an important parameter that characterizes the charge condition on the surface of particles, which can directly reflect the nature of particle surface charges. h-BN adsorbs more OH^−^ than H^+^ when its dispersed in water and the overall display is negative thanks to the pH of water is greater than the isoelectric point of it (4.3).^48^ As shown in Fig. S2,† the potential of the PEI aqueous solution is positive because the N in the amino group from the PEI can combine with water to ionize H^+^ to form –NH_3_^+^, which leading to the negative charge on h-BN is neutralized.^28,49^

**Fig. 3:**
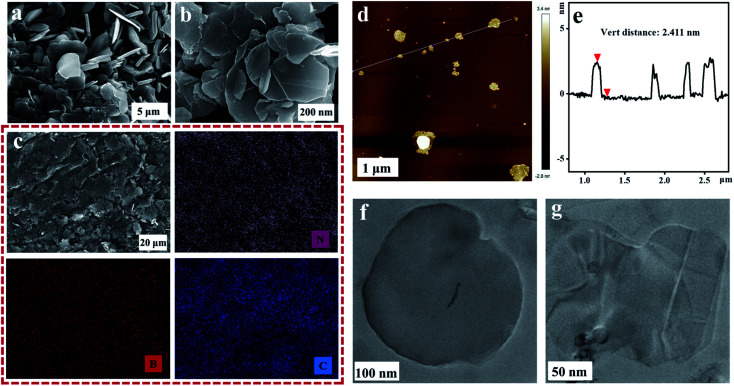
SEM images of (a) h-BN and (b) PEI@BNNS. (c) High-magnification elemental mapping of PEI@BNNS. (d) AFM topographic image of PEI@BNNS and (e) a curve of its surface scale. (f and g) TEM images of PEI@BNNS. Inset in (f) is corresponding electron-diffraction pattern of PEI@BNNS.

SEM, AFM and TEM were used to observe the morphology of h-BN and PEI@BNNS. It can be clearly observed from Fig. 3a that the h-BN without peeling treatment is thicker and has a smooth surface, while Fig. 3b shows the thickness of the PEI@BNNS become thinner compared with former. The reason is that during the sanding process, h-BN first produces a large number of defects, and then the exposed boron atoms on the BN will be grafted from the PEI, which further promotes the peeling of h-BN. X-ray energy spectroscopy (EDS) analysis was also performed on PEI@BNNS due to characterize the microscopic morphology. It can be observed in the Fig. 3c that the samples not only contain B and N elements belonging to boron nitride, C elements from PEI have also been added. According to AFM topographic images in Fig. 3d and e, the lateral and thickness size of PEI@BNNS were about 300 nm and 2.4 nm, corresponding to a thickness of about seven to eight layers. In order to further explore the morphology of h-BN after stripping, TEM tests were performed. Fig. 3g shows that BNNS are transparent, flexible and can be folded. The electron diffraction pattern of the PEI@BNNS in Fig. S3† shows the typical six-fold symmetry,^50^ indicating that the stripped BNNS retain the structural integrity of h-BN. Beyond that, it can be seen from the Fig. 3f and g that the lateral size of the PEI@BNNS is about 300 nm, which is consistent with the AFM results.

### 3.2 Characterizations of TPU composites

**Fig. 4:**
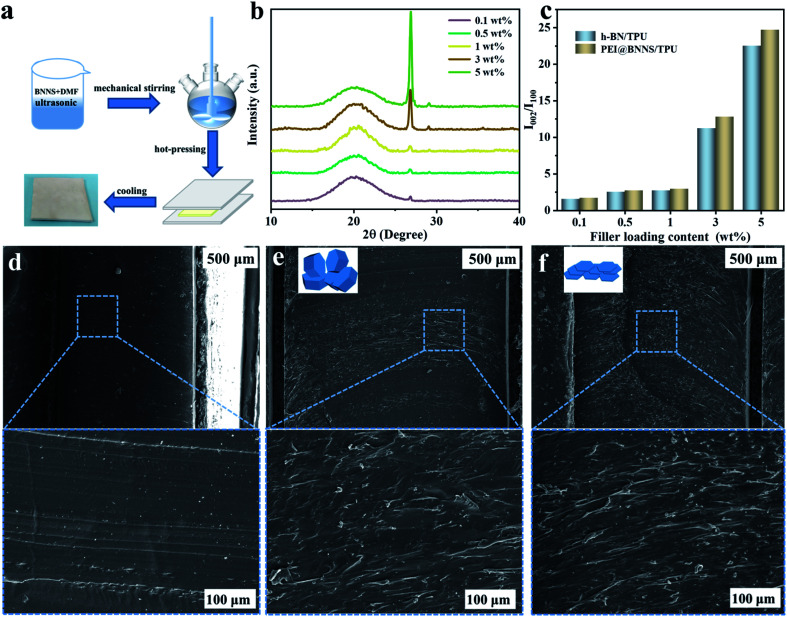
(a) Preparation process of composites. (b) XRD patterns of PEI@BNNS/TPU composites. (c) The orientation degree of h-BN/TPU and PEI@BNNS/TPU composites indicated by the intensity ratio *I*_002_/*I*_100_. SEM images of (d) TPU. (e) 0.1 wt% h-BN/TPU. (f) 0.1 wt% PEI@BNNS/TPU. The insets in (e) and (f) correspond to the state of the filler in TPU, respectively.

TPU composites were prepared by solution blending and hot-pressing with different contents of h-BN and PEI@BNNS, respectively, and the diagram of preparation process was shown in Fig. 4a. XRD analysis was carried out to investigate the orientation of h-BN and PEI@BNNS in TPU matrix.^51^ The characteristic diffraction peaks of h-BN at 2*θ* = 26.5° and 41.6° correspond to the (002) crystal plane and (100) crystal plane, respectively, and their intensity ratio (*I*_002_/*I*_100_) can profile the orientation trend of h-BN and PEI@BNNS in TPU matrix.^52^ As shown in Fig. 4b, c and S4,† the orientation trend keep increasing with the increase of h-BN and PEI@BNNS contents. Noted that, PEI@BNNS/TPU has higher trend than that of h-BN/TPU, which probably due to the flatter shape of PEI@BNNS. To further observe the arrangement of h-BN and PEI@BNNS in TPU matrix, the cross-section SEM images of TPU composites are exhibited in parts (d)–(f) of Fig. 4, respectively. It is show that, pure TPU is neat and smooth, while the cross-section of both TPU composites is rough and uneven. By comparison, PEI@BNNS presents a more orderly arrangement in TPU on account of its higher orientation trend.

**Fig. 5:**
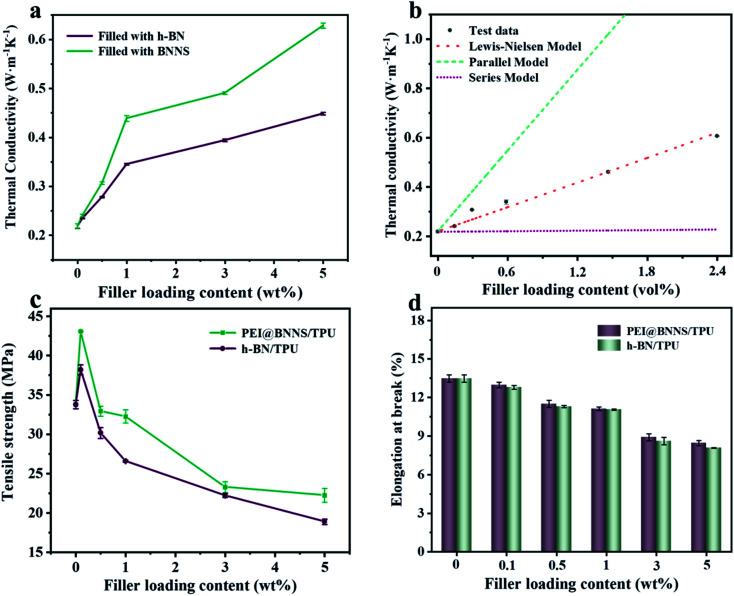
(a) The in-plane thermal conductivity of composites as a function of filler content. (b) Prediction models of thermal conductivity. Tensile strength (c) and elongation at break (d) of composites as a function of filler content.

The *k* of the TPU composites was characterized to discuss the influence of different fillers and different contents. As we can see, *k* of TPU composites increases monotonically with the increase of the filler content in Fig. 5a, which indicates that the fillers in contact with each other reduce the thermal resistance of the interface so that more heat can be passed along the path of the heat conduction network.^53,54^ Besides, the *k* of pure TPU is only 0.22 W m^−1^ K^−1^, while the *k* of 5 wt% h-BN/TPU composite is 0.45 W m^−1^ K^−1^, and that of 5 wt% PEI@BNNS/TPU composites is 0.61 W m^−1^ K^−1^, which are 2 times and 3 times of pure TPU, respectively. The higher *k* of PEI@BNNS/TPU composites can be attributed to the high orientation of PEI@BNNS/TPU composites, which leads to a more completed heat conduction network.^55^

At present, many theoretical, experimental and semi-experimental thermal conductivity models have been proposed to predict the effective thermal conductivity about two-component composites.^56–59^ For two-component composites, the simplest heat conduction model is to consider whether the filler component is parallel to or in series with the heat flow, which will give the upper and lower limits of the thermal conductivity of the composites. The parallel model is:^60^

1 *k*_c_ = *ϕ*_f_*k*_f_ + *ϕ*_m_*k*_m_

The series model is:2
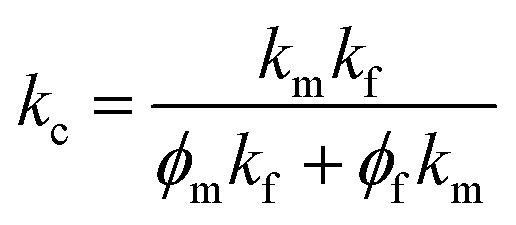
*ϕ*_f_ represent the volume fraction of the filler, *ϕ*_m_ is the maximum volume fraction, *k*_c_, *k*_f_, *k*_m_ represent the thermal conductivity of the composite material, filler, and matrix, respectively. The parallel and series heat conduction models in Fig. 5b respectively show the upper and lower limits of the thermal conductivity of the PEI@BNNS/TPU composite. Taking into account the thermal conductivity, shape, aspect ratio and other factors of the filler itself, the Halpin–Tsai equation modified by Lewis–Nielsen^61^ can better fit the data of the entire concentration range. The equation is:3
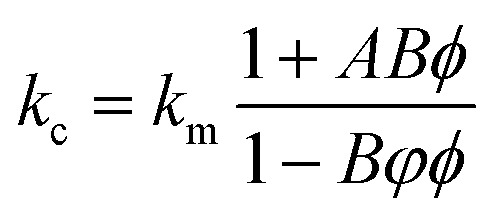
4
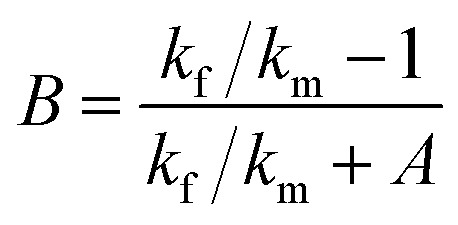
5
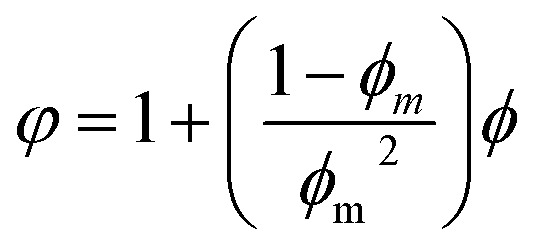
*A* is a constant related to Einstein coefficient, *ϕ*_m_ is the maximum packing fraction of the filler, and *φ* is a function related to the maximum packing fraction *ϕ*_m_ of the filler. For the platelet filler particles in this article, *A* and *ϕ*_m_ are 43.73 and 0.907, respectively.^62^ Compared with the experimental data, the Lewis–Nielsen equation can fit the thermal conductivity of PEI@BNNS/TPU composites relatively well. To characterize the electrical properties of TPU composites, volume resistivity and surface resistivity are used to investigate. As shown in Fig. S5,† the resistivity of TPU composites are all higher than 10^8^ Ω cm, in other words, the composites still maintaining high electrical insulation performance, which can meet the requirements of electronic components.^63^

The effect of fillers on mechanical properties of TPU composites were tested by tensile test, and the optical picture of the tensile test process was shown in Fig. S6.† As shown in Fig. 5c and d, the tensile strength and elongation at break of pure TPU is 33.76 MPa and 13.48%, respectively. Compared with pure TPU, the tensile strength of 0.1 wt% PEI@BNNS/TPU increased from 33.76 MPa to 43.08 MPa, an increase of 27.6%. This is due to the external force is transferred from TPU chain to the internal PEI@BNNS leading to the local stress concentration is weakened, which improving the tensile strength of PEI@BNNS/TPU. In addition, in the case of the same addition amount, the tensile strength and elongation at break of PEI@BNNS/TPU composites are better than that of h-BN/TPU. This result stems from BNNS with PEI grafted-on has better compatibility with TPU, at the same time, flake-shaped PEI@BNNS can help form a thermal network more easily.

In order to further explore the interaction between PEI@BNNS and TPU, we obtained the performance of the storage modulus (*E*′) and loss factor (tan *δ*) of the composite material as a function of temperature through DMA. As shown in Fig. S7a,† the *E*′ values of all composite materials are higher than that of pure TPU and gradually increases with the increase of PEI@BNNS content. One of the reasons is that the hydrogen bond between TPU and PEI@BNNS weakens the liquidity of TPU molecular chain, which causes an improvement in the *E*′ of TPU composites.^62,64^ The value of the peak loss factor (tan *δ*_m_) is usually used to express the glass transition temperature (*T*_g_). As we expected, *T*_g_ increases with the increase of the filling amount in Fig. S7b.† This result shows that, as the content of PEI@BNNS increases, the hydrogen bonding is also increasing, and the interaction prevents the migration of TPU molecular chains and increases the stiffness of the system.^50,65,66^

**Fig. 6:**
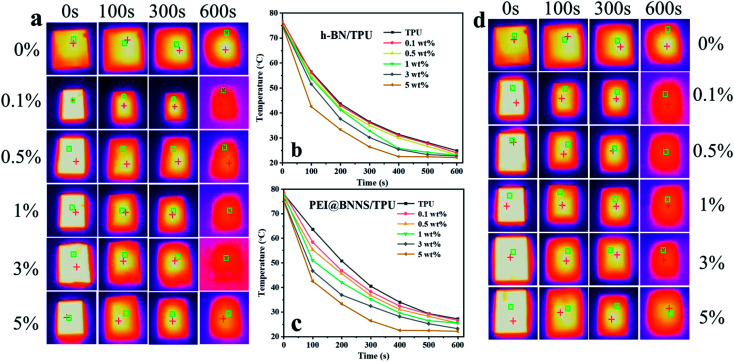
(a) Infrared thermal images and (b) surface temperature variation with cooling time for TPU and h-BN/TPU. (c) Infrared thermal images and (d) surface temperature variation with cooling time for TPU and PEI@BNNS/TPU.

In order to demonstrate the potential applications of the as-prepared TPU composites in thermal management, the infrared thermal imager was used to record the surface temperature of TPU composites during the heat dissipation process with time. Firstly, place all samples in an oven at 80 °C for 6 h to ensure that the sample temperature is uniform, and then transfer to a thermal insulation foam box at room temperature, meanwhile, use a thermal imager to record the temperature change of TPU composites. As shown in Fig. 6, the steady-state temperatures of h-BN/TPU and PEI@BNNS/TPU are 550 s and 450 s, respectively. This is because PEI@BNNS can be arranged more orderly in the TPU matrix than that of h-BN, so that a more effective thermal network can be constructed.

## 4 Conclusion

In summary, h-BN was successfully exfoliated into PEI@BNNS with a transverse size of around 300 nm and a thickness of around 2.5 nm by the mechanical liquid-phase sanding method. A series of PEI@BNNS/TPU and h-BN/TPU composites were prepared by hot-pressing, and finally the effects of different fillers and filler contents on the thermal and mechanical properties of the composites were studied. The results show that the in-plane thermal conductivity of PEI@BNNS/TPU composites with insulating property can reach 0.61 W m^−1^ K^−1^ when PEI@BNNS content is up to 5 wt%, which because disc-liked shape of PEI@BNNS with an excellent radius–thickness ratio can form a more complete heat conduction network.

## Author contributions

X. W. Qu conceived and designed the project. B. Wang performed the experiments, analyzed the data, and wrote the manuscript. H. F. Ji and X. J. Zhang reviewed and modified the manuscript. All authors discussed the results and commented on the manuscript.

## Conflicts of interest

There are no conflicts to declare.

## Supplementary Material

RA-011-D1RA05878A-s001
