# Supplementary material for: Preparation of boron nitride nanosheets via polyethyleneimine assisted sand milling: towards thermal conductivity and insulation applications
Source: RSC Adv. 2021 Nov 29;11(61):38374–82. doi: 10.1039/d1ra05878a (PMC9044043; doi:10.1039/d1ra05878a)
Supplement: RA-011-D1RA05878A-s001 [file RA-011-D1RA05878A-s001.pdf]

Electronic Supplementary Material (ESI) for RSC Advances.
This journal is © The Royal Society of Chemistry 2021

Supporting information for

Preparation of boron nitride nanosheets via polyethyleneimine assisted sand milling: Towards thermal conductivity and insulation application

Bing Wang, Haifeng Ji, Xiaojie Zhang*, Xiongwei Qu*

Hebei Key Laboratory of Functional Polymers, Department of Polymer Materials and Engineering, Hebei University of Technology, 8 Guangrong Street, Tianjin 300130, P. R. China

Email: xwqu@hebut.edu.cn (X. Qu), zhangxj@hebut.edu.cn (X. Zhang)

X-ray photoelectron spectroscopy (XPS) Measurements

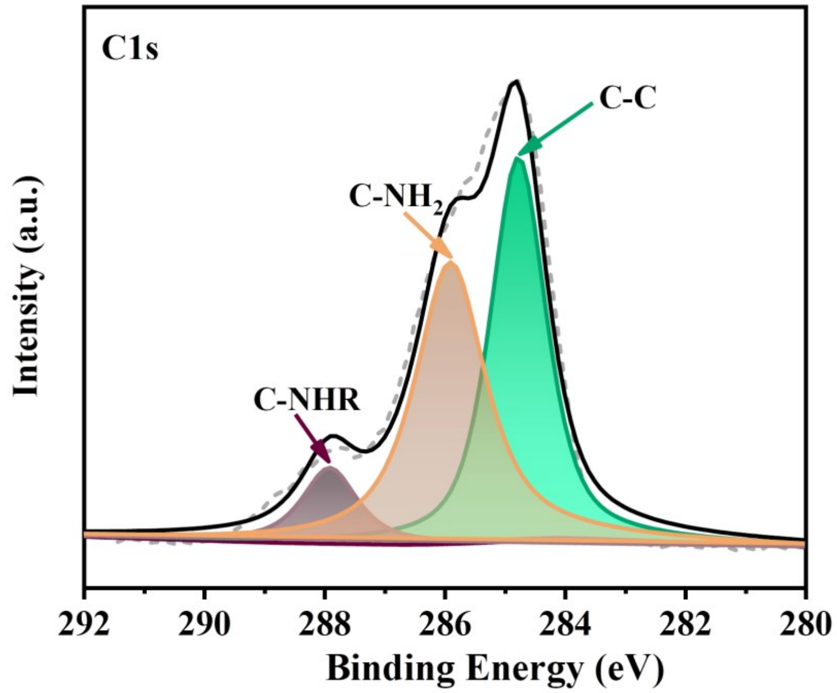

Figure S1 XPS spectra of PEI@BNNS with the high resolution C1s peak-fitting curves.

Zeta Potentials

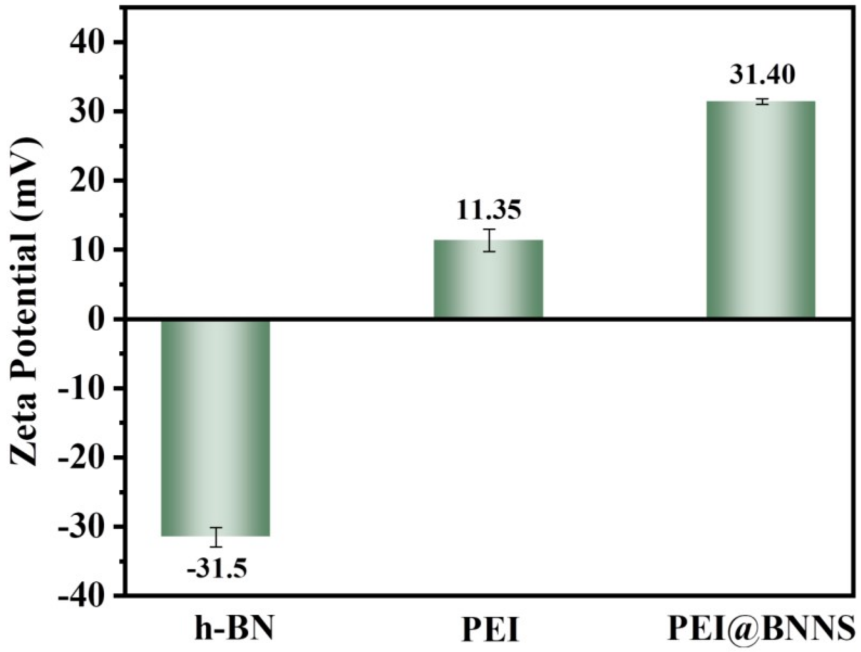

Figure S2 Zeta potential of h-BN, PEI and PEI@BNNS.

Electron diffraction pattern of the PEI@BNNS

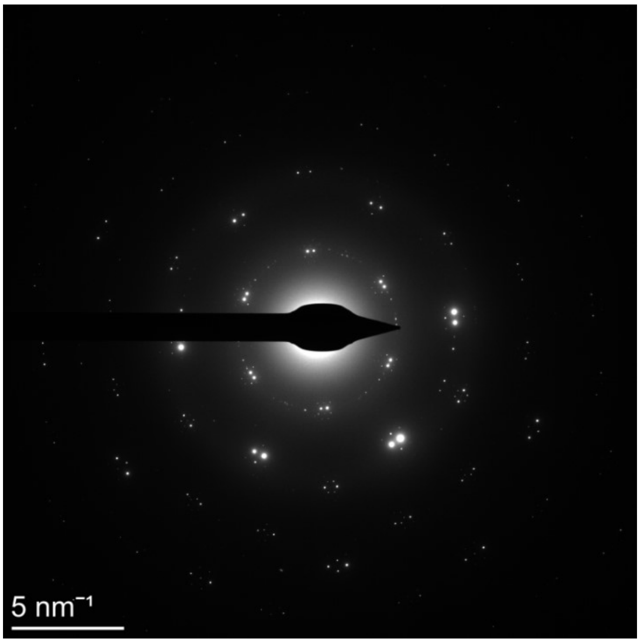

Figure S3 Electron diffraction pattern of the PEI@BNNS.

X-ray diffraction (XRD) Measurements

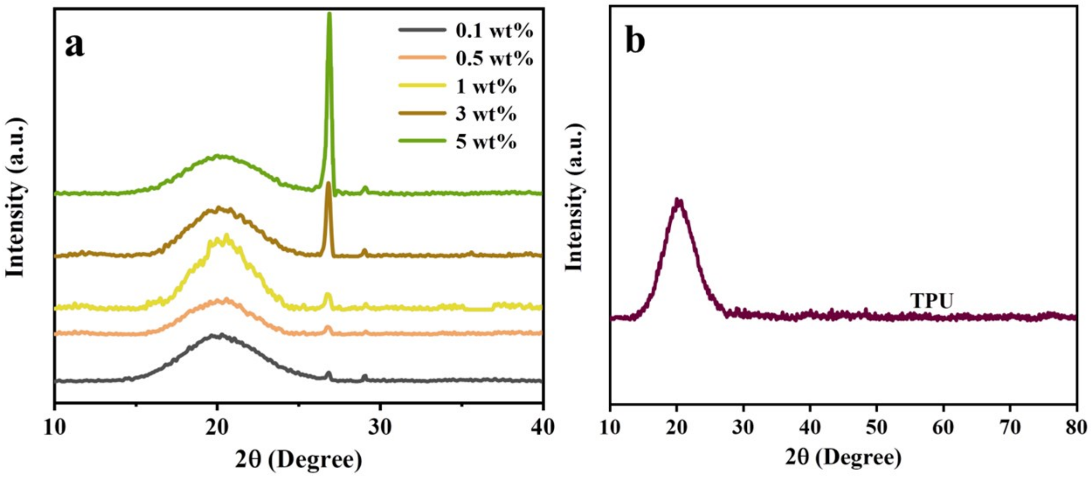

Figure S4 XRD patterns (a) h-BN/TPU composites with different filler loadings. (b) TPU.

Resistivity Measurements

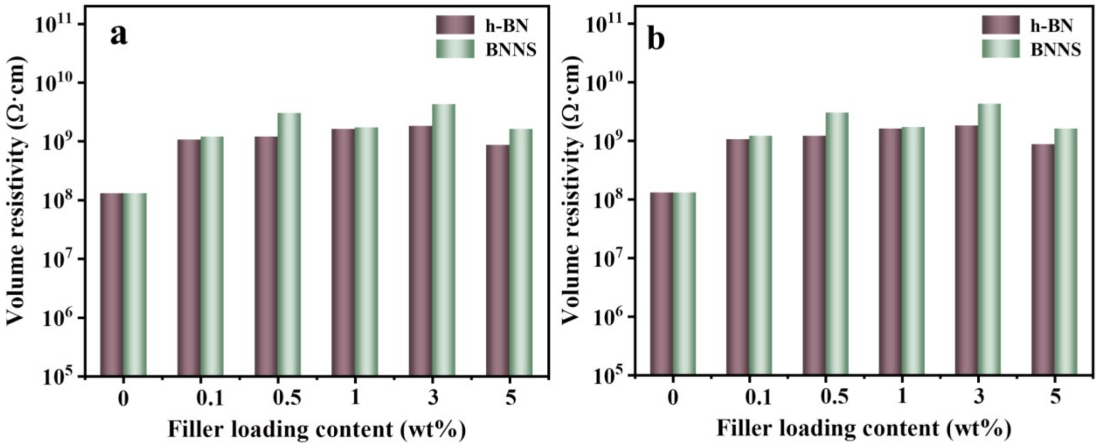

Figure S5 Volume resistivity (a) and surface resistivity (b) of neat TPU, h-BN/TPU and PEI@BNNS/TPU composites.

Tensile strength measurements

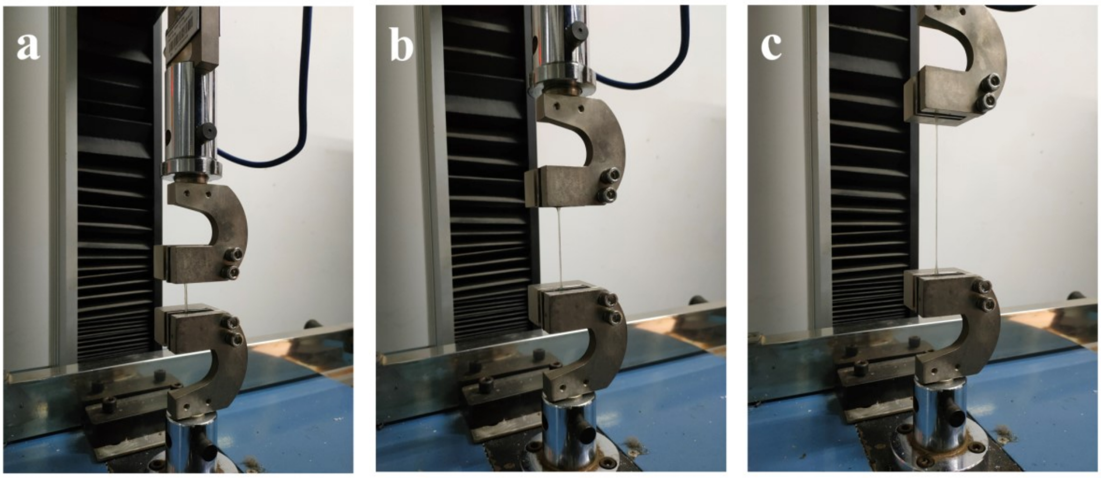

Figure S6 (a)-(c) 5 wt% PEI@BNNS/TPU composite tensile strength test process.

Dynamic Mechanical Analysis

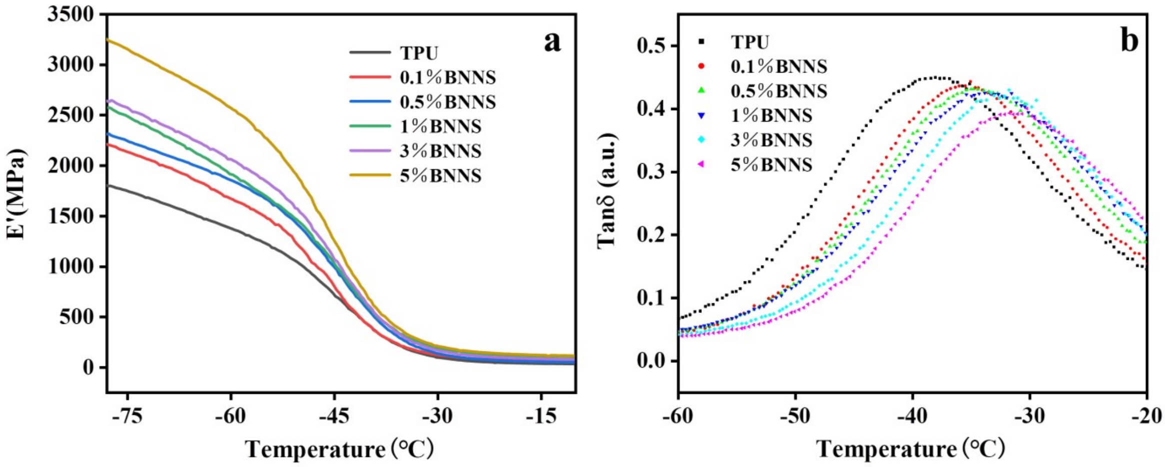

Figure S7 (a) Dynamic mechanical analysis storage modulus (E') and (b) loss factor (tan δ) curves of TPU composites.
